# Association Between Blood Cadmium Levels and Heart Failure Risk: Insights From NHANES 2009–2014

**DOI:** 10.1155/cdr/3656561

**Published:** 2025-10-30

**Authors:** Yong He, Hongkun Wu, Yongjin Luo, Xin Wen, Hao Chen

**Affiliations:** Cardiovascular Surgery, Chongqing General Hospital, Chongqing University, Chongqing, China

**Keywords:** blood cadmium, environmental pollution, heart failure, smoke

## Abstract

**Background:**

Heart failure (HF) is a major global public health problem, and identifying modifiable environmental risk factors is important for prevention. We evaluated the association between blood cadmium levels and the risk of HF in US adults.

**Methods:**

We analyzed data from the National Health and Nutrition Examination Survey (NHANES) 2009–2014. A total of 10,542 adults with complete information on blood cadmium and HF status were included. Weighted multivariable logistic regression models were used to assess the relationship between blood cadmium and self-reported HF. Restricted cubic spline (RCS) regression and two-piecewise linear models were applied to explore potential dose–response relationships.

**Results:**

A cohort of 10,542 US adults from the NHANES 2009–2014 dataset was analyzed, including 321 individuals diagnosed with HF. In fully adjusted, weighted logistic regression models, elevated blood cadmium levels were significantly correlated with an increased prevalence of HF. Specifically, each 1 *μ*g/L increment in blood cadmium was associated with a 42% higher likelihood of HF (odds ratio [OR] = 1.42; 95% confidence interval [CI]: 1.18–1.72; *p* < 0.001). Participants in the highest quartile of blood cadmium levels exhibited more than a threefold increase in the odds of HF compared to those in the lowest quartile (OR = 3.15; 95% CI: 1.81–5.50; *p* < 0.001), with a significant linear trend observed across quartiles (*p* < 0.001). RCS analysis demonstrated a monotonic positive relationship, with an apparent inflection point at approximately 0.13 *μ*g/L, beyond which the risk continued to escalate. This association was generally consistent across various subgroups but was notably stronger among smokers (*p* for interaction < 0.05).

**Conclusions:**

Elevated blood cadmium is an independent risk factor for HF in the United States. These findings suggest that reducing cadmium exposure may be a novel strategy for HF prevention. Prospective studies are warranted to clarify causal relationships and underlying mechanisms.

## 1. Introduction

Cardiovascular disease (CVD) is one of the leading causes of morbidity and mortality worldwide and contributes substantially to the global disease burden and healthcare costs [1]. Heart failure (HF), as a major clinical outcome of CVD, affects over 64 million people globally and is associated with high rates of hospitalization, disability, and healthcare expenditures [2]. Identifying risk factors for HF is therefore crucial for early prevention and management.

In recent years, environmental exposures to heavy metals have gained attention for their adverse effects on the cardiovascular system. Cadmium is a ubiquitous environmental pollutant that enters the body via smoking, dietary intake, and industrial emissions. It accumulates over time and can induce oxidative stress, chronic inflammation, and endothelial dysfunction, thereby negatively impacting cardiovascular health [3]. From a mechanistic perspective, the accumulation of cadmium in cardiac tissue, particularly within the left ventricular myocardium, has been shown to directly induce cellular damage, a conclusion corroborated by numerous studies. Cadmium, recognized as a heavy metal pollutant, has been confirmed to exert toxic effects across various cell types. Empirical evidence indicates that cadmium exposure triggers a cascade of cellular damage mechanisms, including oxidative stress, inflammatory responses, and apoptosis [4, 5]. However, the relationship between blood cadmium (BCD) and HF has not been extensively studied, especially in large, nationally representative samples.

Moreover, the effects of cadmium on cardiovascular health may vary by population subgroups, such as differences in race, sex, education, or lifestyle factors [6]. Therefore, a systematic evaluation of BCD and HF risk, including exploration of dose–response relationships and subgroup differences, could provide insights into potential mechanisms and inform targeted prevention strategies. In this study, we leveraged data from the National Health and Nutrition Examination Survey (NHANES) 2009–2014 to investigate the association between BCD levels and HF risk among US adults. We employed multivariable logistic regression models to assess the odds ratio (OR) for HF associated with cadmium and used restricted cubic spline (RCS) and threshold analysis to explore possible nonlinear dose–response effects.

## 2. Materials and Methods

### 2.1. Data Source and Study Population

**Figure 1:**
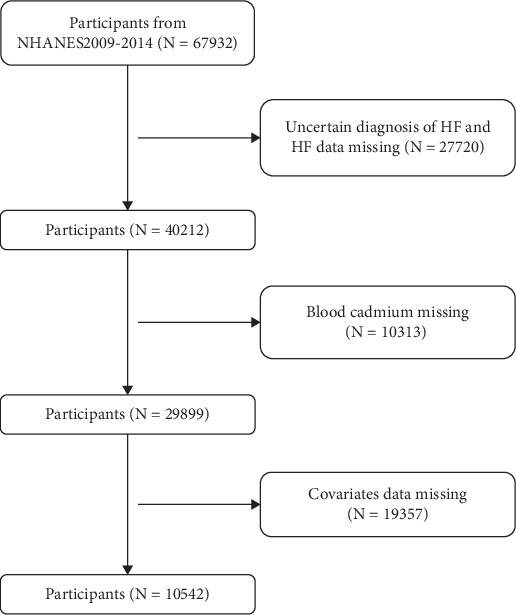
Flowchart of participant selection.

This cross-sectional study utilized data from the 2009–2014 cycles of the NHANES, a continuous nationally representative survey of the civilian, noninstitutionalized US population (https://www.cdc.gov/nchs/nhanes). All participants provided informed consent, and the NHANES protocol was approved by the NCHS Research Ethics Review Board. Among 67,932 total NHANES participants in 2009–2014, we excluded those with missing data on HF status, BCD, or covariates. Participants with uncertain HF diagnosis were also excluded. Ultimately, 10,542 adults (≥ 18 years old) with complete data were included in the analysis (Figure 1).

### 2.2. Definition of HF

HF was defined based on participants' self-reported medical history. Specifically, participants were asked: “Has a doctor or other health professional ever told {you/SP} that {you/s/he} had congestive heart failure?” A positive response was taken to indicate a history of HF.

### 2.3. Covariates

Covariates included demographic factors (age, sex, race/ethnicity, marital status, education level, and income), lifestyle factors (smoking and alcohol use), clinical variables (body mass index [BMI]), comorbidities (hypertension, diabetes mellitus, kidney failure, and anemia), and laboratory measures (creatinine, blood urea nitrogen, and blood lead). Race/ethnicity was categorized as Mexican American, other Hispanic, non-Hispanic White, non-Hispanic Black, or other. Continuous covariates (year, BMI, creatinine, and blood urea nitrogen) were treated as continuous variables, while others were categorical. Definitions and coding were consistent with NHANES protocols.

### 2.4. Statistical Analysis

All analyses were performed using survey weights to account for the complex NHANES sampling design. Baseline characteristics were summarized by HF status (Figure 1). Continuous variables are presented as mean ± standard deviation (SD) and categorical variables as counts and percentages. Differences between HF and non-HF groups were evaluated using *t*-tests or chi-square tests as appropriate.

We used ORs to estimate the association between BCD levels and HF risk. BCD was analyzed both as a continuous variable and as categorized into quartiles. Three models were constructed: Model 1 was unadjusted; Model 2 was adjusted for age, sex, and race/ethnicity; and Model 3 was further adjusted for education, income, BMI, hypertension, diabetes, kidney failure, smoking, drinking, creatinine, blood urea nitrogen, anemia, and blood lead. Adjusted ORs and 95% confidence intervals (CIs) were reported. We also conducted a test for linear trend by including cadmium as a continuous variable across quartiles.

RCS models were applied to assess potential nonlinearity in the dose–response relationship between BCD and HF risk. A two-piecewise linear regression (threshold analysis) was further performed to identify any inflection point and estimate separate slopes below and above the threshold. The likelihood ratio test compared one-line versus two-line models to determine significance of nonlinearity. All analyses were performed in R 4.4.1, and statistical significance was set at two-sided *p* < 0.05.

## 3. Results

### 3.1. Baseline Characteristics

A total of 10,542 participants (mean age 48.99 years, 50% male) were included, of whom 321 had HF and 10,221 did not. As shown in Figure 1, compared with the non-HF group, the HF group was older (mean age 66.19 vs. 48.45 years, *p* < 0.001), had a higher proportion of men (56% vs. 50%, *p* = 0.037), and had a higher prevalence of risk factors such as hypertension, diabetes, and smoking. The HF group also had higher average BMI and lower family income. Mean BCD was significantly higher in the HF group than the non-HF group (0.67 ± 0.67 *μ*g/L vs. 0.51 ± 0.58 *μ*g/L, *p* < 0.001). Other variables are summarized in Table 1. These baseline comparisons suggest that HF participants had more adverse cardiovascular risk profiles.

### 3.2. Association Between BCD and HF

We evaluated the association between BCD and HF using logistic regression across three models (Model 1: unadjusted; Model 2: adjusted for demographic variables; and Model 3: adjusted for all covariates). When modeled as a continuous variable, higher BCD was consistently associated with greater odds of HF in all models: Model 1, OR 1.39 (95% CI 1.23–1.57; *p* < 0.001); Model 2, OR 1.41 (95% CI 1.21–1.63; *p* < 0.001); and Model 3, OR 1.42 (95% CI 1.18–1.72; *p* < 0.001). In quartile analyses (Q1 as reference), odds of HF increased across categories: Model 1 (unadjusted): Q2 2.23 (1.34–3.71; *p* = 0.003), Q3 4.13 (2.32–7.35; *p* < 0.001), and Q4 5.50 (3.22–9.40; *p* < 0.001); Model 2 (demographic adjusted): Q2 1.55 (0.92–2.61; *p* = 0.10), Q3 1.85 (0.98–3.50; *p* = 0.05), and Q4 2.79 (1.63–4.77; *p* < 0.001); and Model 3 (fully adjusted): Q2 1.92 (1.12–3.28; *p* = 0.02), Q3 2.12 (1.10–4.08; *p* = 0.02), and Q4 3.15 (1.81–5.50; *p* < 0.001). Tests for linear trend across cadmium quartiles were significant in all models (*p* for trend < 0.001), indicating a robust dose–response relationship. While estimates for intermediate quartiles attenuated after adjustment, the overall pattern persisted, and the association for the highest quartile remained strong (Table 2).

### 3.3. Subgroup Analysis

**Figure 2:**
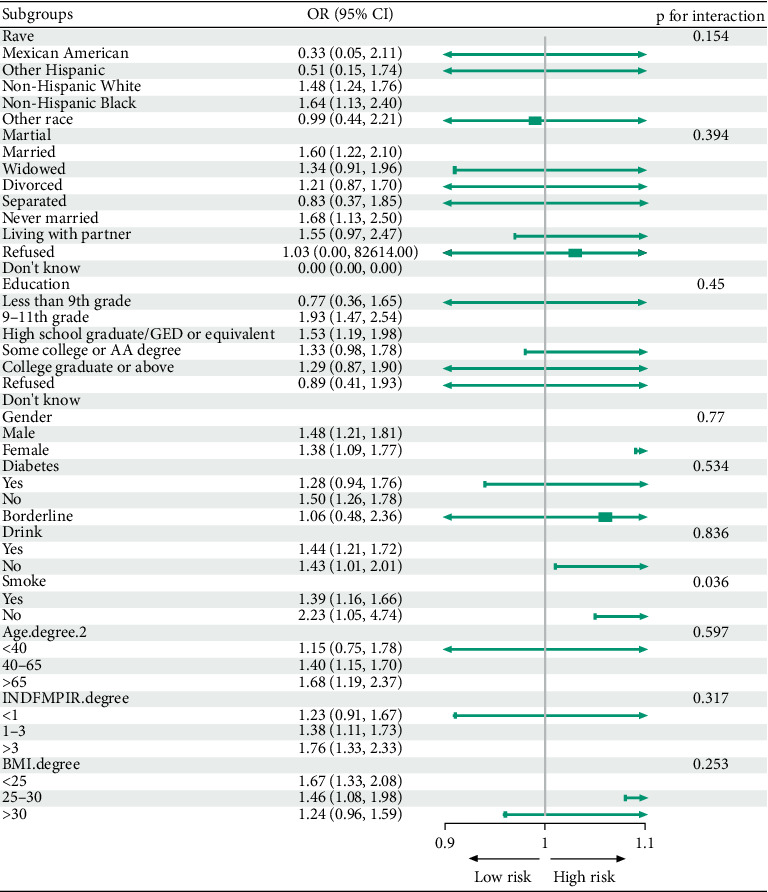
Subgroup analysis of the association between blood cadmium and heart failure.

We conducted subgroup analyses to assess the consistency of the cadmium–HF association across population subgroups (Figure 2). ORs were calculated within strata of age, sex, race/ethnicity, education, smoking status, and comorbidities. The positive association between BCD and HF risk remained generally consistent across most subgroups. The only statistically significant interaction was with smoking status: Smokers exhibited a stronger cadmium–HF relationship than nonsmokers. Other factors (age, sex, race, education, and comorbidities) did not significantly modify the association. These findings suggest that the cadmium–HF link is robust across diverse demographic and clinical backgrounds, though smoking may amplify cadmium's cardiovascular toxicity (Figure 2).

### 3.4. Nonlinear Relationship and Threshold Analysis

**Figure 3:**
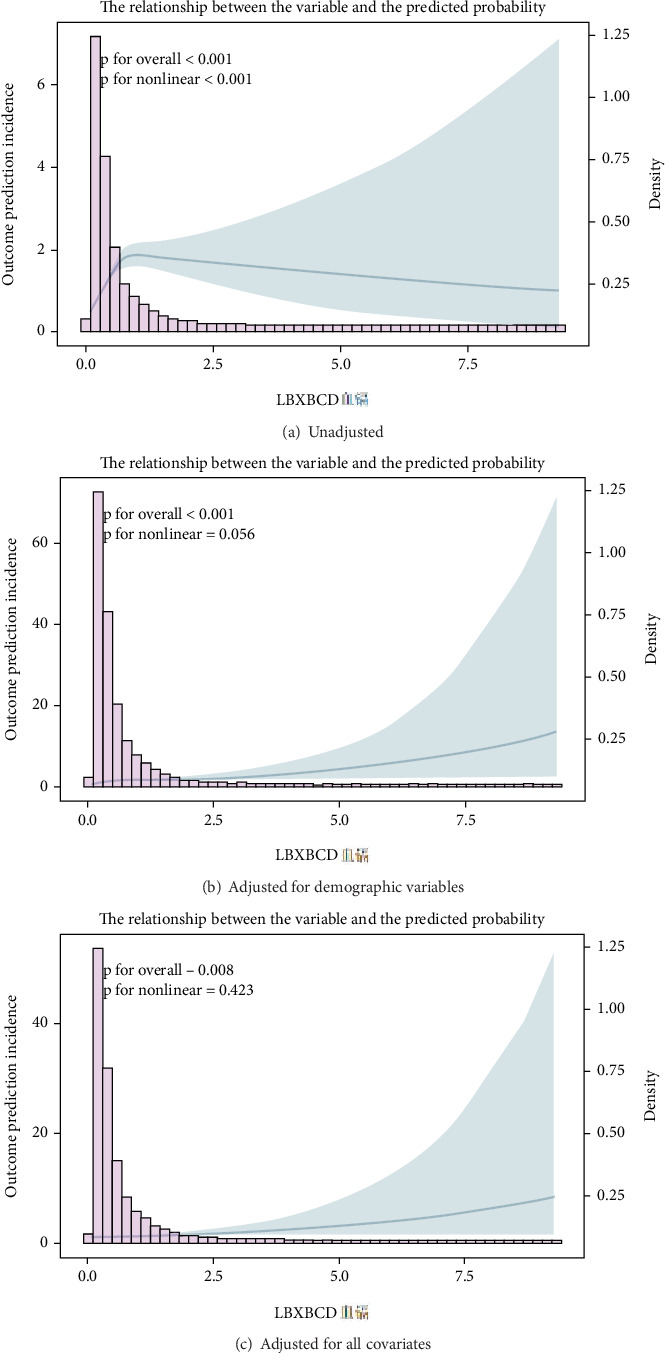
RCS analysis of blood cadmium and heart failure risk.

We employed RCSs to flexibly model the dose–response relationship between BCD levels and the incidence of HF. Three models were constructed (Figure 3). In the unadjusted model (Figure 3a), a significant and positive nonlinear association was observed (*p* for overall < 0.001 and *p* for nonlinearity < 0.001). After adjusting for demographic variables including age, sex, and race (Model 2, Figure 3b), the overall association remained highly significant (*p* for overall < 0.001), while the nonlinear trend was borderline significant (*p* for nonlinearity = 0.056). In the fully adjusted model (Model 3, Figure 3c), which accounted for all potential confounders, including anemia, the relationship between BCD levels and the incidence of HF was most accurately characterized as linear (*p* for overall = 0.008 and *p* for nonlinearity = 0.423). To further investigate this relationship, we compared standard logistic regression with two-piecewise logistic regression. The latter analysis suggested an exploratory inflection point at approximately 0.13 *μ*g/L. However, the likelihood ratio test did not demonstrate a statistically significant improvement in fit for the two-piecewise model (*p* = 0.144), indicating that a linear dose–response relationship sufficiently describes the association (Table 3).

## 4. Discussion

In this nationally representative sample of US adults, we found that higher BCD levels were independently associated with an increased risk of HF. This association persisted after extensive adjustment for demographic factors, lifestyle, comorbidities, and other laboratory measures. The dose–response relationship appeared to be linear, with a threshold at approximately 0.13 *μ*g/L, below which incremental increases in cadmium were associated with a larger rise in HF risk. Subgroup analyses indicated that the cadmium–HF link was generally consistent, although smokers exhibited a stronger association, suggesting that smoking may exacerbate cadmium's cardiovascular toxicity.

Our findings are consistent with previous evidence linking cadmium exposure to adverse cardiovascular outcomes. For example, a Swedish cohort study reported that individuals in the highest quartile of BCD had nearly double the HF incidence compared to the lowest quartile [7]. Recent meta-analyses have shown positive associations between cadmium exposure (in blood or urine) and overall CVD risk, including HF [8–11]. The present study extends this literature by focusing specifically on HF and by demonstrating a linear dose–response in a large, contemporary US population.

Several biological mechanisms may underlie the observed link between cadmium and HF. Cadmium induces oxidative stress by disrupting the balance between reactive oxygen species and antioxidants, leading to cellular injury and apoptosis [12, 13]. It also activates proinflammatory pathways (e.g., NF-*κ*B and NLRP3 inflammasome), resulting in elevated cytokines such as IL-6 and TNF-*α* that can contribute to myocardial damage [4, 12]. Cadmium exposure impairs endothelial function—via effects on Notch signaling and VE-cadherin expression—and can promote atherosclerosis and myocardial fibrosis [14–18]. These multifactorial processes (inflammation, oxidative stress, and endothelial dysfunction) can culminate in ventricular remodeling and HF [19–21]. Our epidemiological results provide human evidence supporting cadmium as an environmental cardiotoxin.

The stronger cadmium–HF association observed in smokers is biologically plausible, since tobacco is a major source of cadmium exposure [22, 23], and smoking itself induces oxidative stress and endothelial damage. Smoking should be regarded as an effect modifier rather than solely a confounder. From a biological perspective, tobacco smoke serves as a significant source of cadmium exposure and concurrently induces oxidative stress and endothelial dysfunction, potentially enhancing cadmium's cardiovascular toxicity through synergistic mechanisms. Statistically, the significant interaction (*p* for interaction < 0.05) corroborates the presence of effect modification, and this interaction is now documented in the figure caption. The interaction suggests that smokers may reach higher body cadmium loads or have synergistic risk [24, 25]. Other subgroup findings (e.g., by race or education) might reflect differences in environmental exposures or susceptibility, though these interactions were not statistically significant.

Previous studies utilizing NHANES data have investigated the relationship between cadmium exposure and cardiovascular outcomes, yet they differ significantly from our study in terms of design, covariate adjustment, and analytical methodologies. For instance, Lin et al. employed NHANES data from 2003 to 2018, utilizing propensity score matching followed by logistic regression to examine HF incidence and Cox regression for mortality outcomes [26]. Their findings indicated a positive association between BCD levels and HF, with an OR of approximately 1.35 (95% CI: 1.05–1.72), and they depicted the dose–response relationship using RCSs, which suggested a largely linear pattern without a distinct threshold. Conversely, Tellez-Plaza et al. concentrated on cardiovascular mortality using NHANES data from 1999 to 2004, applying Cox regression while adjusting for variables such as age, sex, smoking status, kidney function, and other common covariates [27]. Their results demonstrated that individuals in the higher cadmium exposure range (80th vs. 20th percentile) had an elevated hazard ratio for CVD mortality, approximately 1.69 (95% CI: 1.03–2.77). Notably, these prior studies did not specifically evaluate HF incidence, and few accounted for hematologic factors such as anemia. In comparison to previous reports, our study presents several distinct differences. Firstly, our research specifically targets HF incidence, as opposed to the broader CVD outcomes or mortality examined in other studies. Secondly, our fully adjusted model incorporates anemia as a covariate, alongside renal indices, comorbidities, and blood lead levels. The inclusion of anemia significantly altered the spline fit. Thirdly, unlike most prior studies, we conducted a formal test for threshold effects using a two-piecewise logistic model and a likelihood ratio test, which demonstrated that the linear model provided an adequate fit. Lastly, by investigating the effect modification by smoking, we discovered a significant interaction, indicating that tobacco exposure enhances the cardiotoxic effects of cadmium. In summary, these contrasts indicate that although previous studies based on NHANES data identified cadmium as a cardiovascular risk factor, our analysis advances this evidence by specifically examining HF, incorporating additional biological covariates such as anemia, and revealing the instability of the apparent threshold across various model specifications. This reinforces the conclusion that the association between cadmium and HF is most accurately described as linear, with smoking serving as a significant effect modifier.

This study's strengths include the use of a large, nationally representative dataset, standardized biomonitoring for cadmium, and comprehensive covariate adjustment, including coexposures such as blood lead. The application of RCSs allowed us to assess potential nonlinearities, an analytic feature often absent in earlier research.

Several limitations warrant caution. First, the cross-sectional design precludes causal inference. Second, HF diagnosis relied on self-report, which may introduce nondifferential misclassification. Third, residual confounding is possible. Dietary cadmium intake, occupational exposure history, cotinine, COPD status (available only in later cycles), and biomarkers such as fibroblast growth factor-23 were not available for full adjustment. Moreover, COPD status—available only in later NHANES cycles—may confound or mediate the association, as COPD is both a consequence of cadmium exposure and a risk factor for HF [28]. Fourth, a single BCD measurement reflects recent to intermediate exposure, not cumulative lifetime burden; longitudinal monitoring would be superior given cadmium's long half-life. Finally, the relatively small number of HF cases limits power for detecting effect modification, and subgroup findings should be interpreted cautiously.

Despite these caveats, the findings have clear implications. Cadmium exposure is preventable. Reducing environmental emissions, limiting contamination of food chains, and reinforcing tobacco control could meaningfully lower population cadmium burden. Clinically, individuals at elevated risk for HF—especially smokers—may benefit from targeted screening and counseling on exposure sources. The identification of a potential t In the fully adjusted model, was most accurately characterized the cadmium–HF relationship was best characterized as linear, and the likelihood ratio test did not support a nonlinear threshold. Thus, the dose–response association is adequately described by a linear model. hreshold near 0.13 *μ*g/L, while requiring replication, may inform future environmental standards and regulatory limits.

## 5. Conclusion

In summary, elevated BCD is associated with a higher risk of HF among US adults, with evidence of a linear dose–response effect. These findings highlight environmental cadmium exposure as a potential modifiable risk factor for HF. Public health efforts to reduce cadmium pollution and exposure—through smoking cessation, dietary changes, and pollution control—may help mitigate HF risk. Further prospective studies are needed to confirm causality and elucidate underlying biological mechanisms.

## Figures and Tables

**Table 1:** Baseline characteristics of participants by heart failure status (NHANES 2009–2014).

**Characteristic**	**N**	**Overall, ** **N** = 10, 542^**a**^	**H** **F** = 321^**a**^	**Non** − **HF** = 10,221^**a**^	**p** ** value** ^**b**^
Gender	10,542				0.036
Male		5256 (50%)	179 (56%)	5077 (50%)	
Female		5286 (50%)	142 (44%)	5144 (50%)	
Age	10,542	48.99 ± (17.69)	66.25 ± (13.03)	48.45 ± (17.54)	< 0.001
Marital	10,542				< 0.001
Married		5340 (51%)	157 (49%)	5183 (51%)	
Widowed		826 (7.8%)	80 (25%)	746 (7.3%)	
Divorced		1168 (11%)	44 (14%)	1124 (11%)	
Separated		342 (3.2%)	7 (2.2%)	335 (3.3%)	
Never married		2017 (19%)	20 (6.2%)	1997 (20%)	
Living with partner		846 (8.0%)	13 (4.0%)	833 (8.1%)	
Refused		2 (< 0.1%)	0 (0%)	2 (< 0.1%)	
Do not know		1 (< 0.1%)	0 (0%)	1 (< 0.1%)	
Race	10,542				< 0.001
Mexican American		1412 (13%)	19 (5.9%)	1393 (14%)	
Other Hispanic		990 (9.4%)	25 (7.8%)	965 (9.4%)	
Non-Hispanic White		4882 (46%)	194 (60%)	4688 (46%)	
Non-Hispanic Black		2158 (20%)	68 (21%)	2090 (20%)	
Other race		1100 (10%)	15 (4.7%)	1085 (11%)	
Education	10,542				< 0.001
Less than 9th grade		963 (9.1%)	50 (16%)	913 (8.9%)	
9–11th grade		1464 (14%)	61 (19%)	1403 (14%)	
High school graduate/GED or equivalent		2360 (22%)	86 (27%)	2274 (22%)	
Some college or AA degree		3191 (30%)	90 (28%)	3101 (30%)	
College graduate or above		2556 (24%)	34 (11%)	2522 (25%)	
Refused		3 (< 0.1%)	0 (0%)	3 (< 0.1%)	
Do not know		5 (< 0.1%)	0 (0%)	5 (< 0.1%)	
Family income	10,542	2.50 ± (1.65)	1.89 ± (1.35)	2.52 ± (1.65)	0.015
Blood cadmium	10,542	0.52 ± (0.58)	0.67 ± (0.67)	0.58 ± (0.58)	< 0.001
Anemia	10,542				0.905
Yes		353 (3.3%)	11 (3.4%)	342 (3.3%)	
No		10,184 (97%)	310 (97%)	9874 (97%)	
Refused		1 (< 0.1%)	0 (0%)	1 (< 0.1%)	
Do not know		4 (< 0.1%)	0 (0%)	4 (< 0.1%)	
Drink	10,542				0.007
Yes		7799 (74%)	216 (67%)	7583 (74%)	
No		2743 (26%)	105 (33%)	2638 (26%)	
BMI	10,542	29.13 ± (6.92)	31.99 ± (8.39)	29.04 ± (6.85)	0.009
Hypertension	10,542				< 0.001
Yes		3781 (36%)	256 (80%)	3525 (34%)	
No		6761 (64%)	65 (20%)	6696 (66%)	
Diabetes	10,542				< 0.001
Yes		1259 (12%)	129 (40%)	1130 (11%)	
No		9034 (86%)	181 (56%)	8853 (87%)	
Borderline		249 (2%)	11 (4%)	238 (2%)	
Kidneys failing	10,542				< 0.001
Yes		308 (3%)	55 (17%)	253 (2%)	
No		10,234 (97%)	266 (83%)	9968 (98%)	
Blood lead	10,542	1.55 ± (1.70)	1.99 ± (1.64)	1.53 ± (1.70)	< 0.001
Creatinine	10,542	0.91 ± (0.47)	1.34 ± (1.07)	0.90 ± (0.43)	< 0.001
Blood urea nitrogen	10,542	13.20 ± (5.89)	20.03 ± (12.09)	12.99 ± (5.45)	< 0.001
Smoke	10,542				< 0.001
Yes		4760 (45%)	186 (58%)	4574 (45%)	
No		5778 (55%)	135 (42%)	5643 (55%)	
Refused		1 (< 0.1%)	0 (0%)	1 (< 0.1%)	
Do not know		3 (< 0.1%)	0 (0%)	3 (< 0.1%)	

*Note: p* values are from *t*-test or chi-square test comparing HF versus non-HF groups.

^a^ *n* (%); mean ± (SD).

^b^Fisher's exact test; Fisher's exact test for count data with simulated *p* value (based on 2000 replicates).

**Table 2:** Multivariable logistic regression analysis of blood cadmium and heart failure.

**Blood cadmium**	**Model 1 OR (95% CI)**	**p**	**Model 2 OR (95% CI)**	**p**	**Model 3 OR (95% CI)**	**p**
Q1	1.39 (1.23, 1.57)	< 0.001	1.41 (1.21, 1.63)	< 0.001	1.42 (1.18, 1.72)	< 0.001
Q2	2.23 (1.34, 3.71)	0.003	1.55 (0.92, 2.61)	0.10	1.92 (1.12, 3.28)	0.02
Q3	4.13 (2.32, 7.35)	< 0.001	1.85 (0.98, 3.50)	0.05	2.12 (1.10, 4.08)	0.02
Q4	5.50 (3.22, 9.40)	< 0.001	2.79 (1.63, 4.77)	< 0.001	3.15 (1.81, 5.50)	< 0.001

**Table 3:** Threshold effect analysis of blood cadmium and heart failure risk.

**Outcome**		**The effect size, 95% CI, ** **p** ** value**
d1	Model 1: Fitting model by standard linear regression	1.32 (1.09–1.55) 0.002
d2	Model 2: Fitting model by two-piecewise linear regression	
d3	Inflection point	0.13
d4	< 0.13	0 (0–409.70) 0.119
d5	> 0.13	1.33 (1.11–1.58) 0.001
d6	*p* for likelihood ratio test	0.14

*Note:* Results are odds ratios (OR) per 1 *μ*g/L increase in cadmium within each segment, with 95% CIs and *p* values.

Abbreviations: CI, confidence interval; OR, odds ratio per 1 *μ*g/L increase in blood cadmium.

## Data Availability

The datasets presented in this study can be found in online repositories. The names of the repository/repositories and accession number(s) can be found at https://www.cdc.gov/nchs/nhanes/.
